# Substitutional Doping for Aluminosilicate Mineral and Superior Water Splitting Performance

**DOI:** 10.1186/s11671-017-2192-8

**Published:** 2017-07-14

**Authors:** Yi Zhang, Liangjie Fu, Zhan Shu, Huaming Yang, Aidong Tang, Tao Jiang

**Affiliations:** 1 School of Minerals Processing and Bioengineering, Central South University, Changsha, 410083 China; 2 Peter A. Rock Thermochemistry Laboratory and NEAT ORU, University of California Davis, One Shields Avenue, Davis, CA 95616 USA; 3 Key Laboratory for Mineral Materials and Application of Hunan Province, Central South University, Changsha, 410083 China; 4 School of Chemistry and Chemical Engineering, Central South University, Changsha, 410083 China

**Keywords:** Aluminosilicate mineral, Halloysite nanotubes, La doping, Photocatalytic hydrogen evolution, DFT calculations

## Abstract

Substitutional doping is a strategy in which atomic impurities are optionally added to a host material to promote its properties, while the geometric and electronic structure evolution of natural nanoclay mineral upon substitutional metal doping is still ambiguous. This paper first designed an efficient lanthanum (La) doping strategy for nanotubular clay (halloysite nanotube, HNT) through the dynamic equilibrium of a substitutional atom in the presence of saturated AlCl_3_ solution, and systematic characterization of the samples was performed. Further density functional theory (DFT) calculations were carried out to reveal the geometric and electronic structure evolution upon metal doping, as well as to verify the atom-level effect of the La doping. The CdS loading and its corresponding water splitting performance could demonstrate the effect of La doping. CdS nanoparticles (11 wt.%) were uniformly deposited on the surface of La-doped halloysite nanotube (La-HNT) with the average size of 5 nm, and the notable photocatalytic hydrogen evolution rate of CdS/La-HNT reached up to 47.5 μmol/h. The results could provide a new strategy for metal ion doping and constructive insight into the substitutional doping mechanism.

**Electronic supplementary material:**

The online version of this article (doi:10.1186/s11671-017-2192-8) contains supplementary material, which is available to authorized users.

## Background

Aluminosilicate minerals (e.g., kaolinite [1–3], zeolite [4, 5] montmorillonite [6, 7], and halloysite [8–13]) have been extensively investigated as catalyst support materials because they are non-toxic to the environment and abundantly available inexpensively from natural deposits. A number of techniques have been used to enhance the functionalities of support materials, such as polymer coating [14, 15], carbon coating [8], and atomic doping [16–19]. Doped aluminosilicate minerals can form in nature, but their synthesis in a laboratory allow for various properties with the specified dopants [20–23]. Incorporating metal ions into the aluminosilicate layer structure makes the corresponding nanomaterial attractive for various applications, including catalysis [24–26], controlled release of pharmaceuticals [27, 28] as well as lithium ion batteries [29, 30]. Recently, based on density functional theory (DFT) computations, the stability, electronic, and mechanical properties of the nanostructured aluminosilicates, like imogolite, halloysite, and chrysotile, have been revealed [31]. However, the substitutional doping mechanism and the electronic structure evolution of a metal into the aluminosilicates are still ambiguous [32, 33].

Towards the goal of improving our understanding of this mechanism, we designed an efficient doping strategy for one of the representative aluminosilicate minerals (halloysite nanotube [34–36], HNT) through the dynamic equilibrium of a substitutional atom in the presence of saturated AlCl_3_ solution, which contained lanthanum salt. Then, a substitutional atomic doping strategy based on La doping into the HNT structure and the replaced part of the Al atom from the Al–O sheet is presented. Halloysite (HNTs, Al_2_Si_2_O_5_(OH)_4_∙nH_2_O),as a natural clay mineral, contains octahsedral gibbsite Al(OH)_3_ and tetrahedral SiO_4_ sheets, and it also consists of hollow cylinders formed by multiple rolled layers. CdS is an attractive semiconductor material that can convert solar energy to chemical energy under visible-light irradiation. Incorporating CdS nanoparticles into La-HNTs and its corresponding water splitting performance could demonstrate the effects of La doping. The diffusion process of Al^3+^ saturated solution and the alumina sheets from halloysite, the change of crystal shapes and surface structures, and the possibility of enhanced catalytic activity were investigated in detail. The microstructures and morphologies of samples were characterized, and the interfacial structure between CdS and La-HNT were investigated. The photocatalytic hydrogen activity was evaluated and the role of La-HNT for enhancing catalytic activity of CdS/La-HNT was also investigated.

## Methods

### Experimental Section

#### Materials Preparation

Halloysite nanotubes (HNT) were obtained from Hunan, China. All chemicals were analytical grade and used without further purification. HNT were pretreated via emulsion dispersion, filtering, washing with distilled water, and drying for 8 h at 313 K. La-HNT were synthesized by a modified hydrothermal route. An amount of 34.3 g of AlCl_3_ was dissolved into 60 mL deionized water to form AlCl_3_ supersaturated solution, while 3 mmol HNT and 6 mmol La(NO_3_)_3_·6H_2_O were dissolved in deionized water (5 mL), respectively. Then La(NO_3_)_3_·6H_2_O solution and the HNT slurry were added to AlCl_3_ supersaturated solution to form a suspension. The resulting suspension was stirred for 10 min in a polypropylene beaker and sonicated for 10 min to break up aggregations of starting material. The volume was limited into 70 mL (L/S = 70–80). The mixture was transferred into a Teflon bottle(100 mL) and treated under auto-generated pressure without stirring at 373 K for 48 h. The autoclave was naturally cooled to room temperature, and the obtained precipitates were filtered and washed several times with deionized water, and finally dried at 353 K in vacuum (denoted as La-HNT). For comparision, acid-treated HNT synthesized by 1.00 g HNT were dissolved in 250 mL of 6 M HCl solution at 373 K in a water bath. The reaction was carried out in a conical flask for 4 h with constant stirring. The conical flask was naturally cooled to room temperature, and the obtained precipitates were filtered and washed several times with deionized water, and finally dried at 353 K in vacuum (denoted as acid-treated HNT).

CdS/La-HNT were synthesized by using the Successive Ionic Layer Adsorption and Reaction (SILAR) method, 3 mmol La-HNT was dissolved into 50 mL 0.5 M Cd(NO_3_)_2_ ethanol solution for 5 min, rinsed with ethanol, and then dissolved for another 5 min in a 50 mL 0.5 M Na_2_S methanol solution, and rinsed again with methanol. Such an immersion cycle was repeated several times until the desired deposition of CdS nanoparticles was achieved. Then, the obtained precipitates were filtered and washed several times with deionized water, and finally dried at 353 K in a vacuum (denoted as CdS/D-Lax-HNT).

#### Characterization

X-ray photoelectron spectroscopy (XPS) analysis was performed on using a Thermo Fisher Scientific K-Alpha 1063 spectrometer equipped with an Al Ka monochromatic X-ray source. The test chamber pressure was maintained below 10^−9^ mbar during spectral acquisition. The XPS binding energy (BE) was internally referenced to the C 1*s* peak (BE = 284.1 eV). The crystalline phases were identified by XRD analysis using a RIGAKU D/max-2550VB1 18-kW powder diffractometer with Cu Ka radiation (*λ* = 1.5418 Å). The data were collected in the scanning range 2θ = 10–80°, with a scanning speed of 2°/min. FTIR spectra were recorded using a Nicolet 5700 spectrophotometer. The specific surface area was calculated from the nitrogen adsorption isotherms using the Brunauer–Emmet–Teller (BET) equation. Transmission electron microscopy (TEM) images were obtained using a JEOL JEM-200CX instrument equipped with an energy dispersive X-ray spectroscopy (EDS) at an accelerating voltage of 200 kV. (PL) spectrum of the sample was detected on a Hitachi H-4500 fluorescence spectrometer using a Xe lamp as the light source. UV–vis spectra of samples in aqueous solution were obtained using a UV-2400 (Shimadzu Corp., Japan) spectrometer. Solid state ^29^Si and ^27^Al MAS NMR spectra were recorded using a Bruker AMX400 spectrometer in a static magnetic field of 9.4 T at a resonance frequency of 79.49 MHz. The electrochemical analysis was carried out in a conventional three-electrode cell using a platinum-black wire and saturated calomel electrode (SCE) as the counter electrode and reference electrode, respectively. The working electrode was prepared on FTO (fluorine tin oxide) conductor glass. In detail, 20 mg of sample was added into 10 mL ethanol and formed a uniform suspension. As in a standard spin-coating process, the ethanol suspension was spread onto FTO glass, whose side part was previously protected using Scotch tape. The spinning was at a high speed of 150 rps and then dried in an oven at 70 °C for 1 h. The transient photocurrent responses of different samples were measured in 0.1 M Na_2_S + 0.02 M Na_2_SO_3_ aqueous solution under visible-light irradiation (≥420 nm) at 0 V vs. SCE. The illuminated area of the working electrode is 2 cm^2^. The photoelectrochemical experiment was performed using a CHI-660A electrochemical workstation (ChenHua Instruments Co. Ltd., Shanghai, China). The electrochemical impedance spectroscopy (EIS) was measured with a CHI-660A electrochemical workstation (ChenHua Instruments Co. Ltd., Shanghai, China), and the electrolyte consisted of 0.01 mol/L potassium hexacyanoferrate (III), 0.01 mol/L potassium hexacyanoferrate (II), and 0.5 mol/L KCl. The applied potential was open circuit potential (OCP).

#### Photocatalytic Reaction

Water splitting reactions were carried out in a gas-closed circulation within a vacuum. A sample of 100 mg photocatalyst powder was dispersed in a 300-mL aqueous solution of 0.1 M Na_2_S and 0.1 M Na_2_SO_3_. The light source was a 300-W Xe lamp, and the light intensity reaching the surface of the reaction solution was 135 mW/cm^2^. The amount of H_2_ evolution was determined using a gas chromatograph (Agilent Technologies: 6890 N).

#### Computational Details

All calculations were performed with the CASTEP code, based on first-principle density functional theory (DFT). The local density approximation (LDA) potential was used for the calculations. The ultrasoft pseudo-potential plane-wave formalism and an energy cutoff of 400 eV were used. The Monkhorst-Pack grid with 3 × 3 × 1 *k*-points mesh was used for the accurate calculation of DOS results, while Gamma point was used during geometry relaxation. The self-consistent total energy in the ground state was effectively obtained by the density-mixing scheme. For geometry optimizations, the convergence threshold for self-consistent field (SCF) tolerance was set to 1.0 × 10^−6^ eV/atom, all forces on the atoms were converged to less than 0.03 eV/Å, the total stress tensor was reduced to the order of 0.05 GPa, and the maximum ionic displacement was within 0.001 Å. The cell parameters and atomic coordination of the structures were optimized using a Broyden–Fletcher–Goldfarb–Shanno (BFGS) minimization algorithm.

## Results and Discussion

**Fig. 1:**
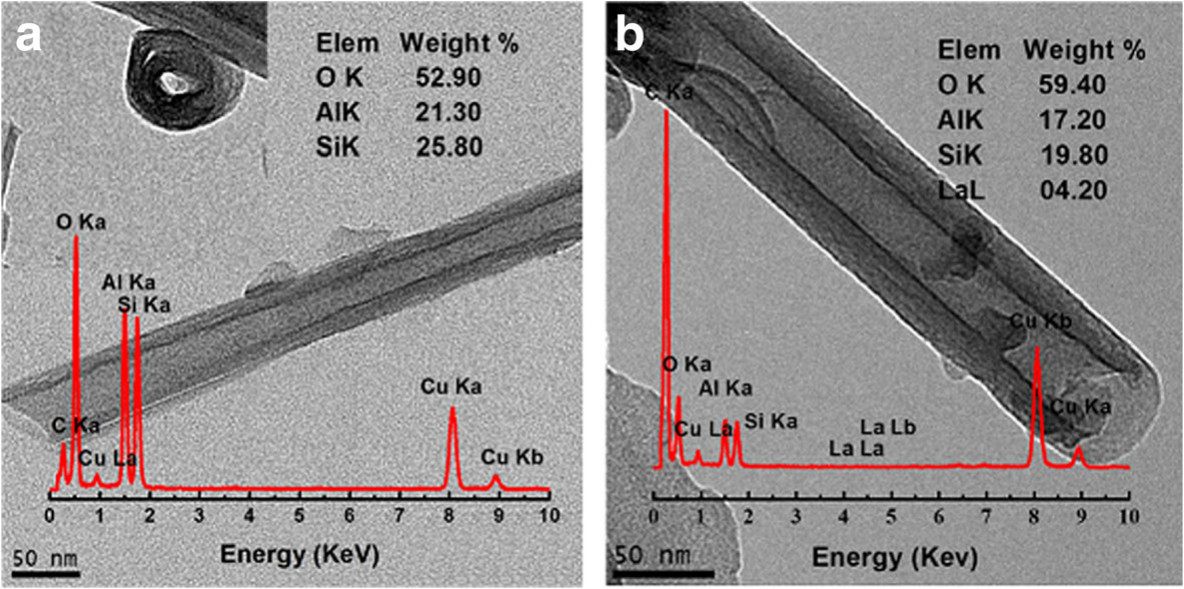
TEM images of **a** HNT and **b** La-HNT samples

The integrity of morphology is important to ensure the success of doping because acid can damage the structure of halloysite while saturated AlCl_3_ solution will not. TEM images of HNT and La-HNT are shown in Fig. 1. HNT shows a multilayer tubular structure 0.7–1.5 μm in length, with a 50–75-nm outer diameter, and a 10–30-nm inner diameter (Fig. 1a). After La doping, the typical tubular morphology has retained with the La content of 4.2% (Fig. 1b). For the acid-treated HNT, the typical tubular morphology was also retained (Additional file 1: Figure S1a), but amorphous.

**Fig. 2:**
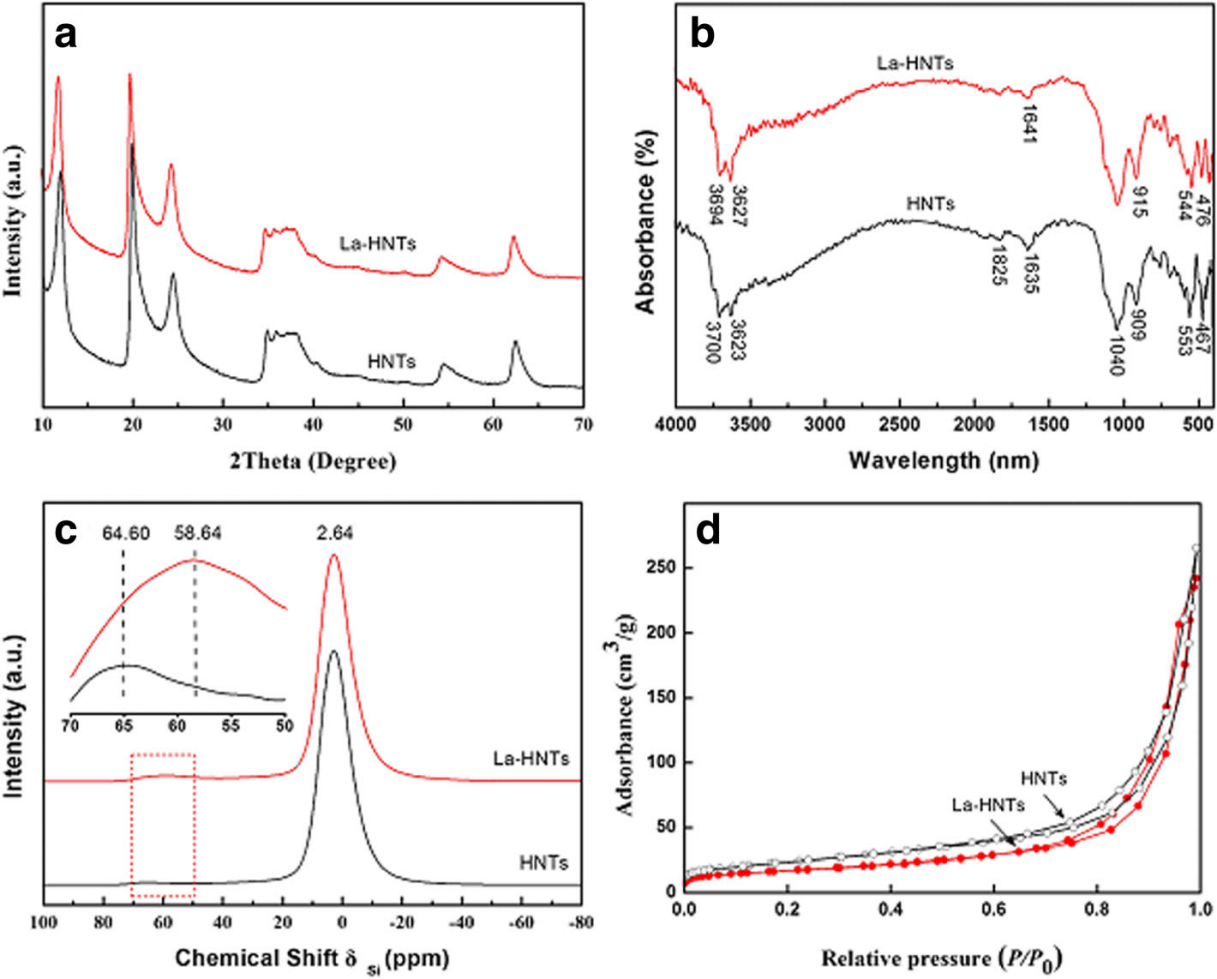
**a** XRD patterns, **b** FTIR spectra, **c** ^27^Al NMR spectra, and **d** nitrogen adsorption–desorption isotherms of HNT and La-HNT samples

The structure of HNT can be influenced by doping due to the larger size of the La atom used to replace the Al atom. For La-HNT, the characteristic data of halloysite (JCPDS card No. 29-1487) can be observed in Fig. 2a and Additional file 1: Table S1, which indicate that the crystalline phase of La-HNT has remained. The slight shift of the first peak in La-HNTs is attributed to the replacement of Al atoms by larger size La atoms, which imparts the enlargement of the (001) interlamellar spacing. However, there is only a broad peak centered at 22° for acid-treated HNT (Additional file 1: Figure S1b), which indicates that there is amorphous silica. The FTIR spectra of HNT and La-HNT are shown in Fig. 2b, and the relative assignment of each vibrational peak are listed in Additional file 1: Table S2. For HNT, these assignments are based on the previous literatures [37–40]. For La-HNT, the O–H deformation vibration of the inner Al–OH groups at 909 cm^−1^ and the Al–OSi deformation vibration of Al–O tetrahedral sheets at 553 cm^−1^ shift to 915 and 544 cm^−1^, respectively. However, the gibbsite octahedral sheet of halloysite is completely destroyed and the Al–O stretching bands disappear for the acid-treated HNT (Additional file 1: Figure S1c). All of these observations prove the successful La doping into the structure of HNT and the change of the HNT structure influenced by La doping. The ^27^Al CP/MAS NMR spectra of HNT and La-HNT samples are presented in Fig. 2c. The resonance signal at −3 ppm is assigned to 6-coordinated Al. The resonance signal at 64 ppm is attributed to the 4-coordinated Al shifting to 58 ppm after La doping, indicating that the environment of the Al atom was influenced by La doping. However, there are three peaks at −111.32, −101.70, and −91.71 ppm in the acid-treated HNT, identified as Si(OSi)_4_, Si(OSi)_3_OH, and Si(OSi)_2_(OH)_2_ (Additional file 1: Figure S1d), respectively. The nitrogen sorption isotherms of HNT and La-HNT are shown in Fig. 2d, and the relative data are summarized in Additional file 1: Table S3. The S_BET_, pore volume, and average pore diameter of La-HNT are 59 m^2^/g, 0.37 cm^3^/g, and 25 nm, respectively. The S_BET_ and pore volume values of La-HNT is lower than that of HNT (82 m^2^/g, 0.41 cm^3^/g), while its average pore diameter (25 nm) is higher than that of HNT (20 nm). However, the S_BET_ value and pore volume for the acid-treated HNT are three times higher than that of HNT (Additional file 1: Figure S1e). All of these results demonstrate that La doping influences on the structure of HNT, given that the S_BET_ for La-HNT is lower than that of HNT, and there is a decrease in total pore volume. The decreased S_BET_ and pore volume could be assigned to the part of halloysite layers collapsed, and the increased pore size could be associated with the Al atom being replaced by the La atom, consistent with the XRD and FTIR analyses [18]. Hence, it is speculated that La-HNT can serve as an excellent support by changing the chemical and structure performance of halloysite, and thus could contribute to the enhanced catalysis activity for HNT-based catalytic nanocomposite materials.

**Fig. 3:**
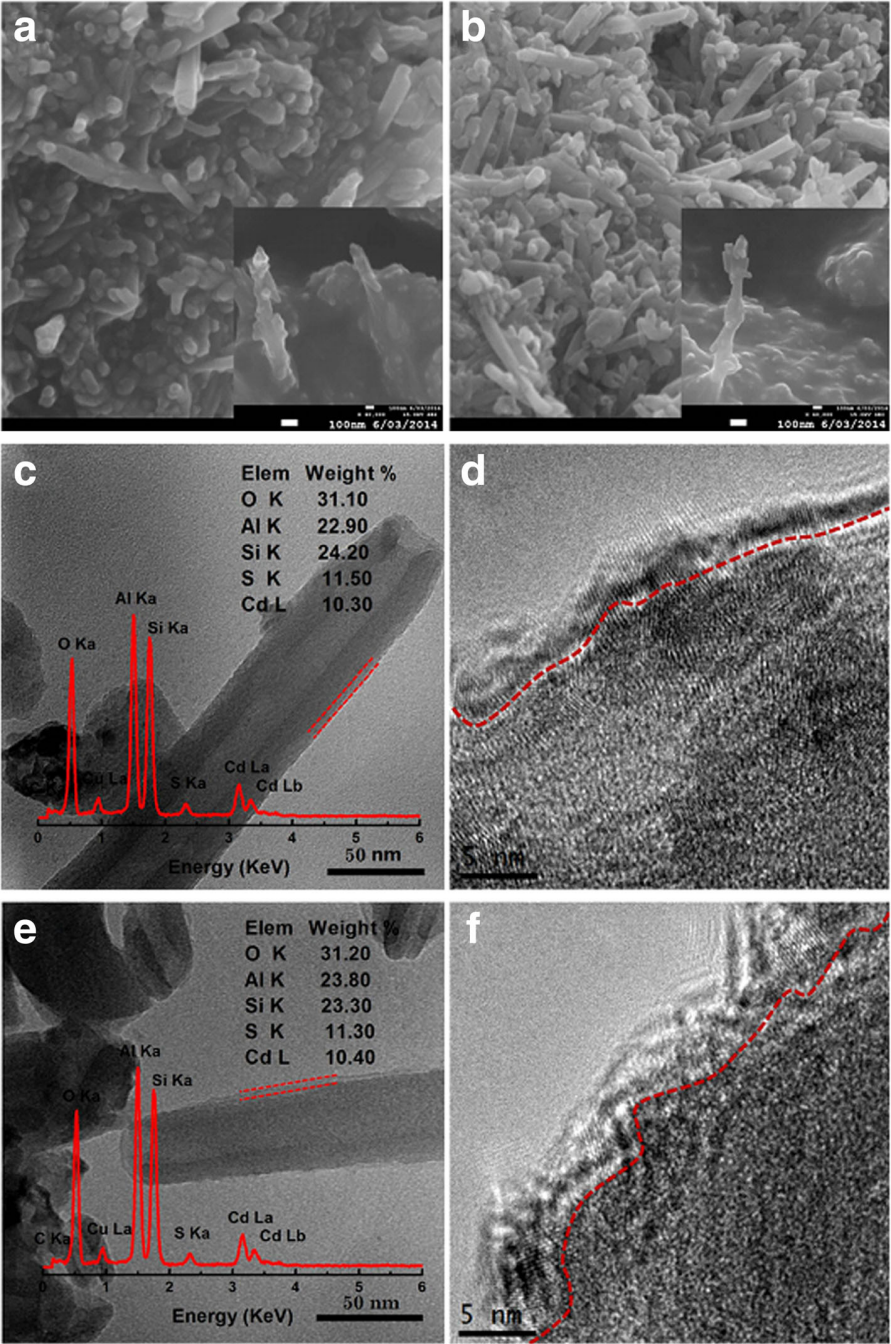
SEM and TEM images of **a**, **c**, **d** CdS/HNT and **b**, **e**, **f** CdS/La-HNT samples

To confirm the effects of La doping, CdS nanoparticles were deposited on the surface of La-HNT and HNT. Figure 3a–c, e clearly shows that the surface of HNT and La-HNT are well covered with a continuous, dense, and uniform CdS nanoparticle layer with a thickness of about 5 nm. However, the La element has not been detected in the CdS/La-HNT sample (inset in Fig. 3e). The disappearance of the La element in the CdS/La-HNTs may be attributed to the shelter of the CdS nanoparticle layer, plus the relevant low La element. The ratio of CdS in CdS/HNT to CdS/La-HNT is 11 wt.%. Figure 3d, f shows a typical HRTEM image of the layer of CdS nanoparticles coating on the surface of the host with a diameter of 5 nm, corresponding with the particle size of 5 nm calculated by Scherrer’s equation (Additional file 1: Figure S2).

**Fig. 4:**
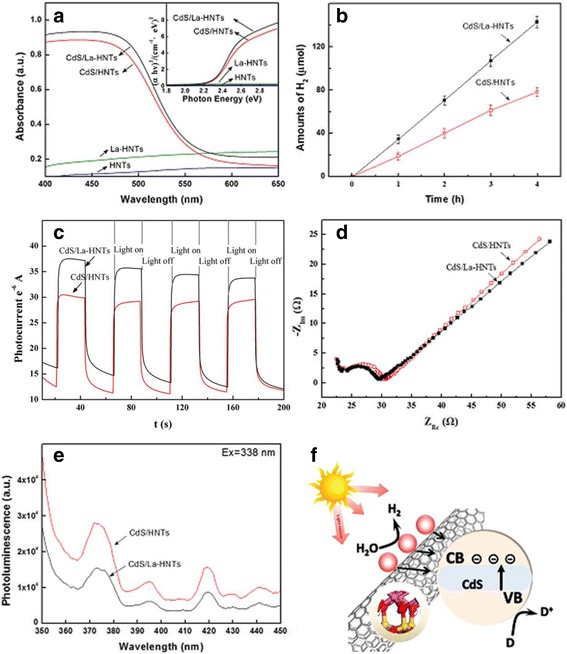
**a** UV-vis spectra, **b** photocatalytic hydrogen curve, **c** the transient photocurrent responses, **d** Nyquist impedance plots, **e** PL spectra, and **f** corresponding catalytic schematic of CdS/HNT and CdS/La-HNT samples

The optical properties of HNT, La-HNT, CdS/HNT, and CdS/La-HNT composites are characterized by UV–vis diffuse reflectance spectroscopy (Fig. 4a). The absorption edges of CdS/HNT and CdS/La-HNT are at 554 and 562 nm, respectively, and CdS/La-HNT shows stronger absorption intensity than CdS/HNT in the visible light region. The La-HNT composites present no absorption in the UV region, and the absorption characteristics are similar to those of HNT in the aspect of wide absorption in the visible light region. The as-obtained *E*_g_ values are 2.31 and 2.25 eV for CdS/HNT and CdS/La-HNT, respectively. The catalytic performance of La-HNT for water splitting was evaluated to reveal how the doping process and corresponding microstructure change affects macroscopic properties. Photocatalytic H_2_ evolution of CdS/HNT and CdS/La-HNT (Fig. 4b) was conducted in an aqueous solution containing SO_3_^2−^ and S^2−^ ions as sacrificial reagents under simulated solar light irradiation [41–43]. The results show that the photocatalytic H_2_ rate of CdS/La-HNT (47.5 μmol/h) is higher than that of CdS/HNT (26.0 μmol/h) under the same reaction conditions (Additional file 1: Table S4), and CdS/La-HNT and CdS/HNT show a higher hydrogenation rate than most of the previously reported CdS-based photocatalysts from the literatures [43–45]. To find out whether the higher photocatalytic H_2_ rate derives from La doping, the transient photocurrent responses of CdS/HNT and CdS/La-HNT were measured in Fig. 4c. It can be clearly seen that the photocurrent rises to a high value rapidly under illumination, and the photocurrent decreases to nearly zero when the light is off. The results show that the photocurrent intensity of CdS/La-HNT is higher than that of CdS/HNT, indicating that more efficient separation of charge carriers had been influenced by La doping.

In order to examine the charge transfer and ion transport, the electrochemical impedance spectroscopy (EIS) was employed [2], and the impedance behavior of CdS/HNT and CdS/La-HNT were measured in Fig. 4d. The Nyquist plots showed a semicircle, which occurred due to the electrochemical process at a high frequency level, followed by a line that indicates the diffusive resistance of the electrolyte and active materials. The semicircle confined charge-transfer resistance, which is closely related to the reversibility of the electrochemical reactions. CdS/La-HNT shows a smaller arc than that of CdS/HNT, indicating that La doping led to more efficient charge transfer over CdS/La-HNT.

The smaller semicircle radius of CdS/La-HNTs compared with CdS/HNTs revealed that CdS/La-HNTs nanocomposite has smaller charge-transfer resistance and has good electrochemical resistance. The effects of La doping are also confirmed by the photoluminescence (PL) emission spectra (Fig. 4e), the lower the PL intensity, the higher the efficiency in photogenerated electron-hole separation.

**Fig. 5:**
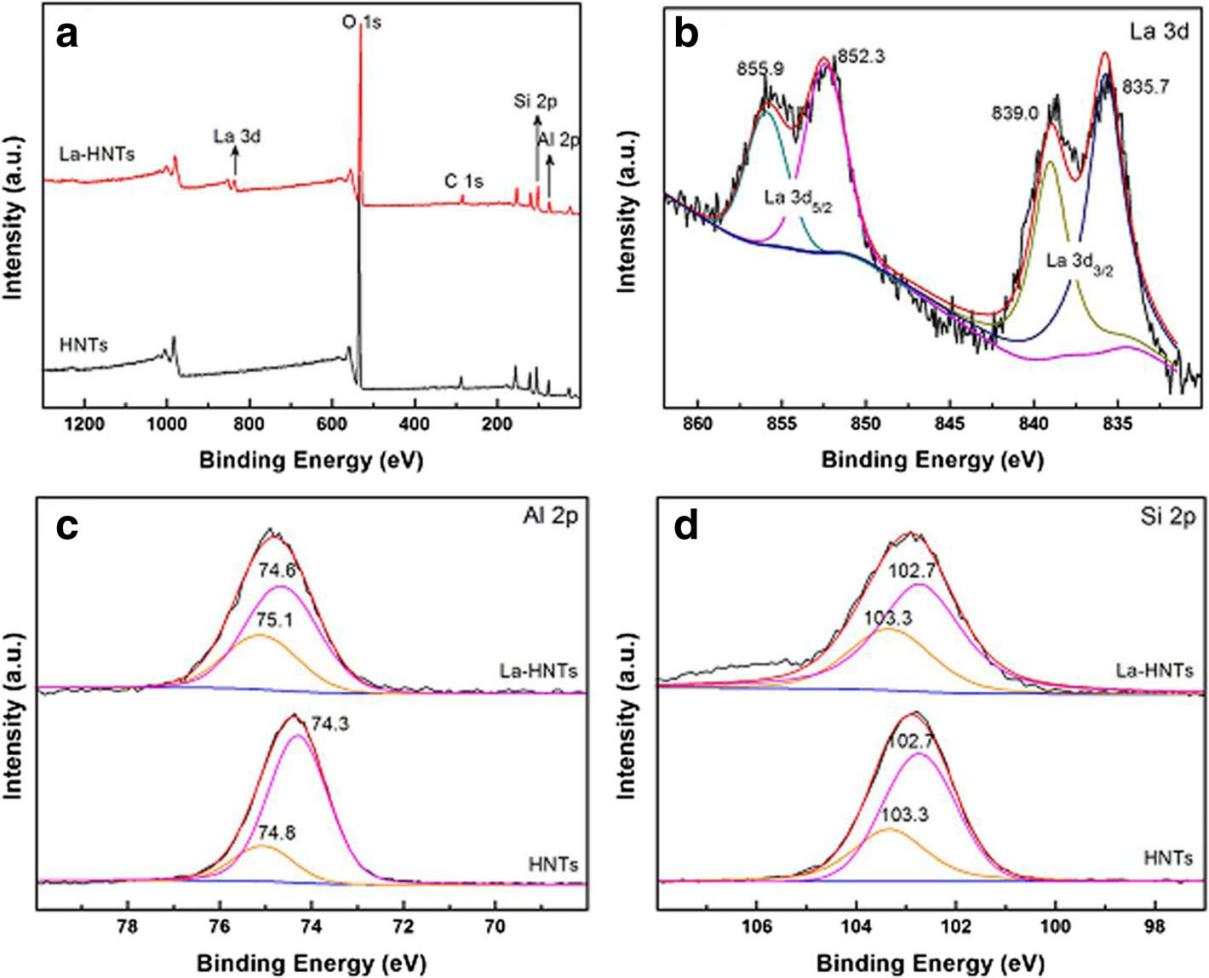
**a** full range, **b** La3d, **c** Al2p and **d** Si2p XPS spectra of HNT and La-HNT samples

Herein, we investigate the surface structure of HNT to further confirm the environment of the Al atom influenced by La doping. The full range of XPS spectra of HNT and La-HNT are exhibited in Fig. 5a, and the relative atomic concentration is shown in Additional file 1: Table S5. Si and Al elements are detected in HNTs and La-HNTs, while La element is detected only in La-HNT. The concentration ratio of Al/Si is 0.62 in La-HNT, lower than that of HNT (0.88), which may be due to the Al atom being replaced by the La atom. For the acid-treated HNT, only Si 2*p* and O 1*s* are detected due to the removal of the alumina sheet (Additional file 1: Figure S1f). Figure 5b shows the La 3*d* spectra, and their peak positions are observed at 835.7 eV (La 3*d*_5/2_), 839.0 eV (La 3*d*_5/2_), 852.3 eV (La 3*d*_3/2_), and 855.9 eV (La 3*d*_3/2_), respectively. Figure 5c, d shows the Al 2*p* and Si 2*p* spectras, and their peak positions are observed at 74.8 eV (Al–OH), 74.3 eV (Al–O), 103.3 eV (Si–OH), and 102.7 eV (Si–O), respectively. Remarkably, the binding energy (BE) values of the Al atom in Al–OH and Al–OSi shifted to 75.1 and 74.6 eV, respectively, while the position of the BE value of Si atoms has not changed. Thus, all of the above results confirm that the La atom replaced part of the Al atom from the Al–O sheet and the environment of Al atoms have been influenced.

**Fig. 6:**
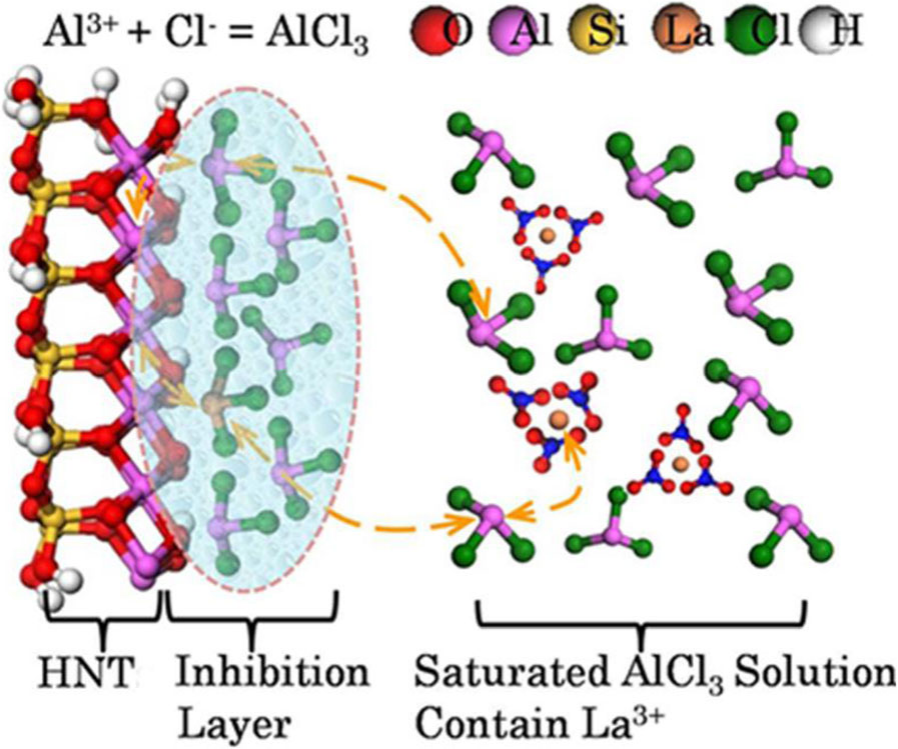
Proposed mechanism of La doping into HNT

Based on the characterization of the La doping into the HNT structure and the replaced part of the Al atom from the Al–O sheet, a substitutional atomic doping model was proposed as follows (Fig. 6). Each layer of HNT consists of a Si tetrahedral sheet and Al octahedral sheet. The distance between the unit cells is difficult to change due to the strong coupling force, so water molecules cannot enter. Thus, halloysite has been identified as the low activity aluminosilicate mineral. In the initial stage, the typical saturated AlCl_3_ solution has been chosen because anhydrous AlCl_3_ is a strong Lewis acid, capable of forming Lewis acid-based adducts with water, such as AlO_2_^2−^, HCl, and H_3_O^+^. Etching starts by the Lewis acid-based adducts interacting with the alumina in the structure of halloysite, and the reaction extent raise with the concentration of Al^3+^ increasing. As the concentration of Al^3+^ is already over saturated, an inhibition layer will be formed, close to the surface of the halloysite. In this situation, cation substitution could occur in the inhibition layer. The La^3+^ ions following Al^3+^ saturated solution are substituted with the soluble aluminum atoms from halloysite in the inhibition layer according to the principle of the concentration of ion saturated solution conservation. Then, the dynamic equilibrium of substitutional atomic doping formed and La doped halloysite composites are achieved. The use of an autoclave in the process was to force the doping process because the radius of La^3+^ ion is larger than that of Al^3+^. The crystal shapes and surface structures can be obviously changed by doping, and the chemical and structural performance of the supporter would be improved, which brings continuous uniformity to nanoparticle loading and improves the catalysis activity for catalytic nanocomposite materials.

**Fig. 7:**
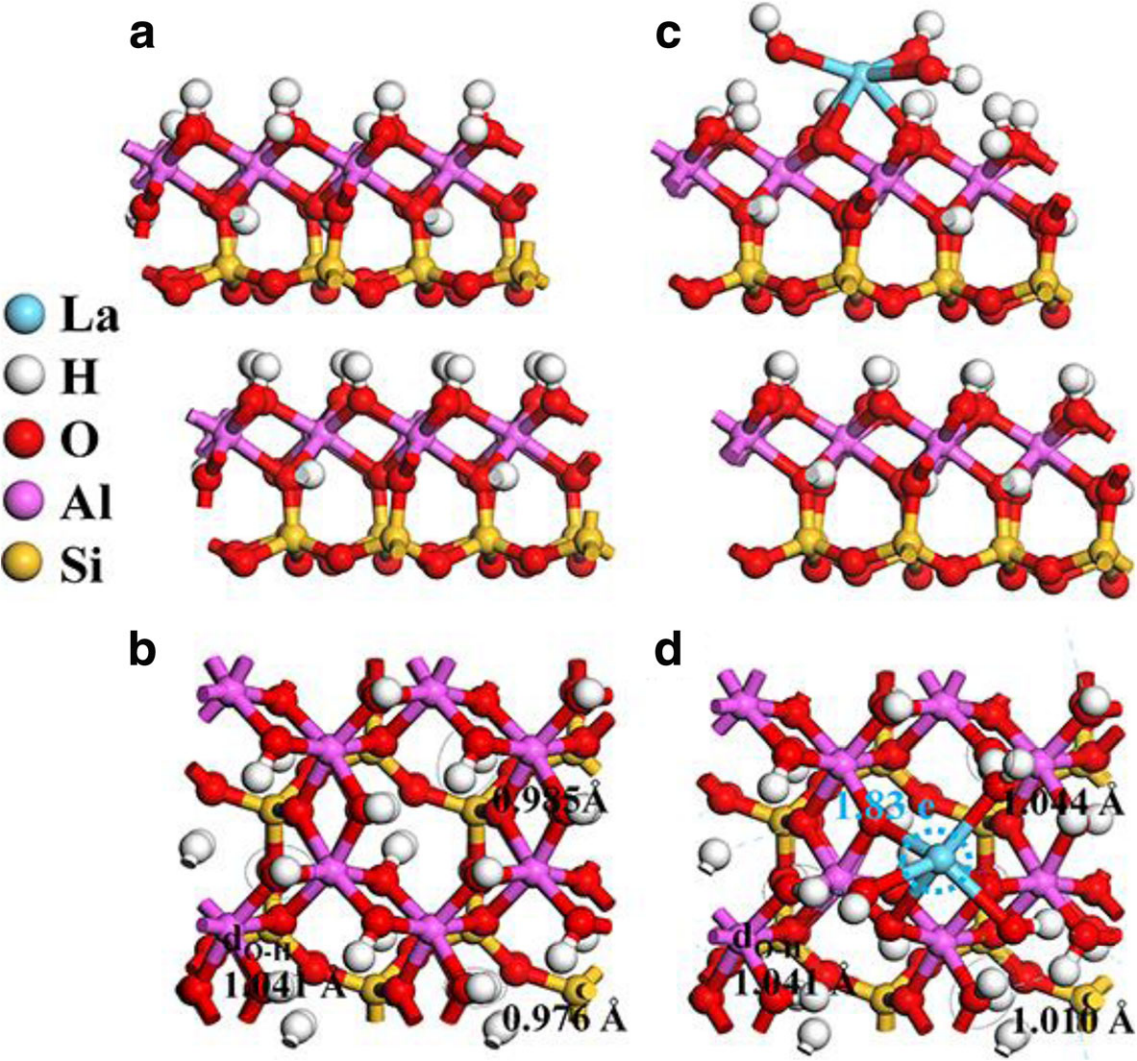
Geometric structure of **a** side view and **b** top view of HNT, and **c** side view and **d** top view of La-HNT

The first-principle density functional theory (DFT) calculations were performed to further investigate the rationality of the doping mechanism and the effect of the La doping. The structure model of HNT is simulated by using crystalline approximations, and the La-HNT is constructed by replacing the Al atom from halloysite with the La atom. The structure of HNT by using kaolinite structure is simulated (Fig. 7a, b), and the surface structures are constructed by cleaving a 2 × 1 layer structure of two Al–O–Si layer thickness out of the bulk halloysite. A vacuum space of 15 Å is used. The La-HNT is constructed by replacing one Al in the Al sheet of halloysite with an La atom or adding La(OH)_3_ on the halloysite surface (Fig. 7c, d). It was found that the Al–O bonds (Al_s_–O) and Si–O bonds perpendicular to the planer layer (Si_ps_–O) on the HNT surface are slightly increased compared to that of HNT bulk structure (Additional file 1: Figure S3), i.e., 0.05–0.10 and 0.02–0.03 Å, respectively. At the same time, the Si–O bonds in the planer layer (Si_is_–O) decreased. Additionally, the O–H bond distribution on HNT surface is broadened due to the surface effect. For the La-doped case, a contraction of the Al_s_–O and Si_ps_–O bonds, as well as an expansion of the Si_is_–O bond, occurs instead after the atomic structure relaxation of La-HNT surface (Additional file 1: Figure S3). The local structure around La shows that the six La–O bonds possess a bond length (around 2.3–2.5 Å) much longer than Al–O bonds, which increases the space around the La doping site (Additional file 1: Figure S4a). Notably, the elongation of three surface OH bonds with H heading directly towards to the O atoms of La(OH)_3_ (H_La_) induces three reactive surface OH groups around the La atom at the La-HNT surface.

**Fig. 8:**
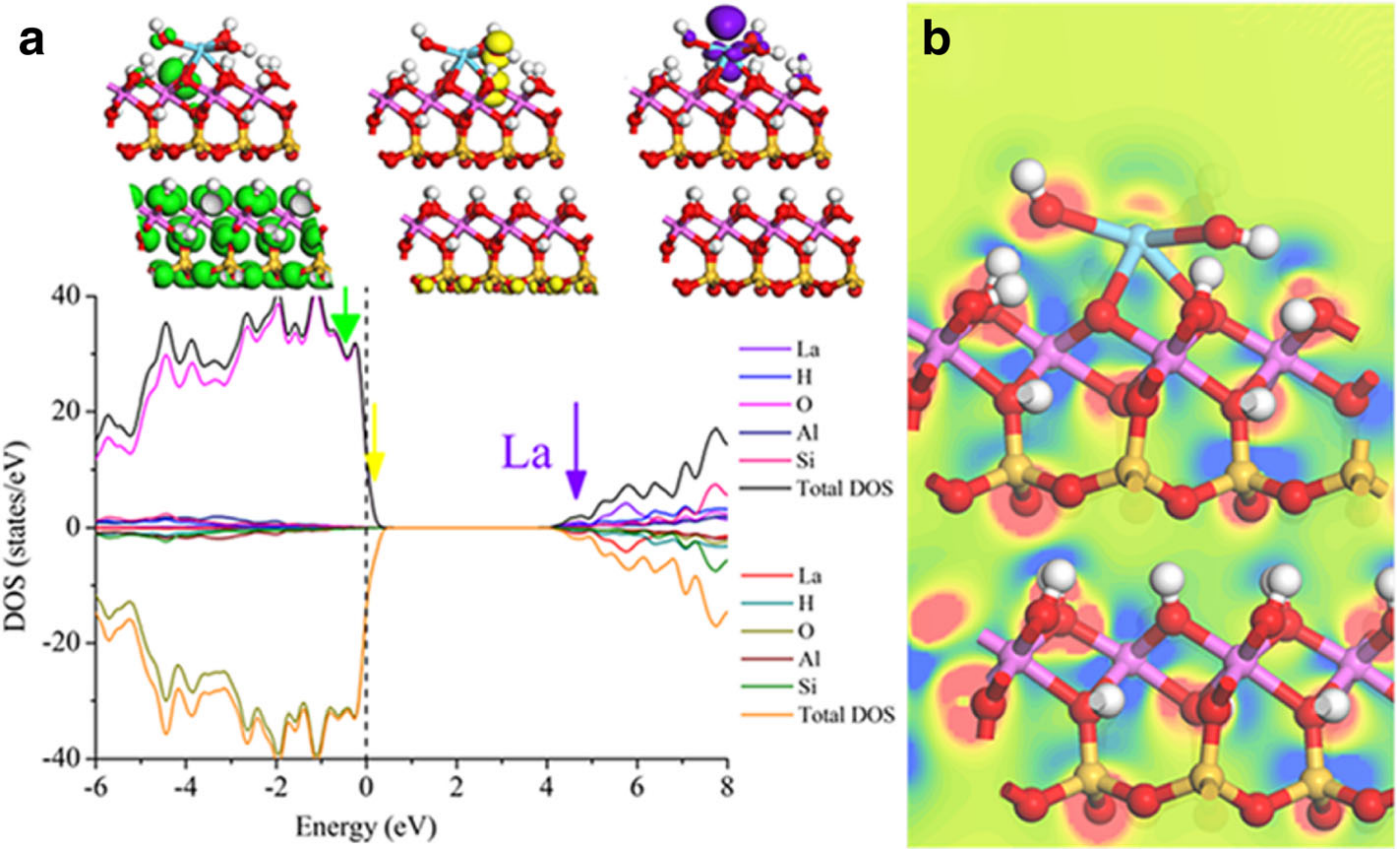
**a** The PDOS results of La-HNT surface. **b** Charge density difference of the La-HNT interface plotted along the plane designated by the *dotted lines* shown in the lower panel of Fig. 7c. *Blue* corresponds to charge depletion and *red* to charge gain. Isosurfaces are shown in the range [−0.08, 0.08] (e/Å^3^). Contours of constant charge density are separated by 0.005 eV/Å^3^

The calculated Mulliken charge for La atom in La-HNT is 1.83 e (Fig. 7d), which corresponds to valance states +3. The PDOS result shows a band gap of 5.2 eV for pristine HNT, and the valance band near the fermi level is mainly composed by O 2*p* state while conduction band minimum (CBM) is mixed by mainly H 1*s*, Si 3*s* 3*p*, and partially O 2*p*, Al 3*s* 3p states (Additional file 1: Figure S4b). The support of La on the HNT surface introduces La 5*d* states in the CBM (Fig. 8a, purple), and the reactive La dopant with 5*d*z^2^ orbitals is confirmed as a donor type impurity. Meanwhile, the three OH groups around La have supplied some lone pair electrons (Fig. 8a, yellow and green) at valance band maximum (VBM), which will enhance the surface absorption ability. The charge density difference around the La-HNT interface shows that there is a charge transfer from the surrounding Al atoms of the HNT surface to La dopants after La doping (Fig. 8b). It is assumed that the La dopants might serve as a charge-transfer bridge between the HNT surface and functional nanoparticles like CdS, and thus the photocatalytic hydrogen evolution rate would be enhanced. Hence, the conformity of the experimentation and simulation has verified the rationality of the simulation model and the substitutional atomic doping model.

## Conclusions

In summary, natural halloysite nanotubes have been successfully doped by La atom. The La doping into the structure of HNT leads to crystal shapes and obvious surface structure changes, which brings continuous uniformity for CdS loading and changes the catalysis activity for nano catalytic composite materials, thus resulting in an enhanced photocatalytic hydrogen evolution rate. The contrast of the photocatalytic hydrogen evolution of CdS/La-HNT and CdS/HNT confirms the high efficiency of the La doping. This result is very encouraging and should be highly applicable for extending the doping technique to other aluminosilicate minerals and the corresponding design of functional materials.

## Additional file

Additional file 1: Supporting Information for Substitutional Doping for Aluminosilicate Mineral and Superior Water Splitting Performance. (DOCX 3550 kb)

## References

[1] Shirzad-Siboni M, Farrokhi M, Soltani RDC, Khataee A, Tajassosi S (2014). Photocatalytic reduction of hexavalent chromium over ZnO nanorods immobilized on kaolin. Ind. Eng. Chem. Res..

[2] Bizaia N, Faria EHD, Ricci GP, Calefi PS, Nassar EJ, Castro KADF, Nakagaki S, Ciuffi KJ, Trujillano R, Vicente MA, Gil A, Korili SA (2009). Porphyrin-kaolinite as efficient catalysts for oxidation reactions. ACS Appl Mater Interfaces.

[3] Yagi M, Narita K (2004). Catalytic O_2_ evolution from water induced by adsorption of [(OH_2_)(Terpy)Mn(μ-O)_2_Mn(Terpy)(OH_2_)]^3+^ complex onto clay compounds. J Am Chem Soc.

[4] Aelst JV, Verboekend D, Philippaerts A, Nuttens N, Kurttepeli M, Gobechiya E, Haouas M, Sree SP, Denayer JFM, Martens JA, Kirschhock CEA, Taulelle F, Bals S, Baron GV, Jacobs PA, Sels BF (2015). Hierarchical zeolite: catalyst design by NH_4_OH treatment of USY zeolite. Adv Funct Mater.

[5] Guo P, Shin J, Greenaway AG, Min JG, Su J, Choi HJ, Liu LF, Cox PA, Hong SB, Wright PA, Zou XD (2015). A zeolite family with expanding structural complexity and embedded isoreticular structures. Nature.

[6] Sun SM, Wang WZ, Jiang D, Zhang L, Li XM, Zheng YL, An Q (2014). Bi_2_WO_6_ quantum dot-intercalated ultrathin montmorillonite nanostructure and its enhanced photocatalytic performance. Nano Res..

[7] Peng K, Fu L, Ouyang J, Yang H (2016). Emerging parallel dual 2D composites: natural clay mineral hybridizing MoS_2_ and interfacial structure. Adv Funct Mater.

[8] Zhang Y, Tang A, Yang H, Ouyang J (2016). Applications and interfaces of halloysite nanocomposites. Appl. Clay Sci..

[9] Zhang Y, Ouyang J, Yang H (2014). Metal oxide nanoparticles deposited onto carbon-coated halloysite nanotubes. Appl. Clay Sci..

[10] Zhang Y, He X, Ouyang J, Yang H (2013). Palladium nanoparticles deposited on silanized halloysite nanotubes: synthesis, characterization and enhanced catalytic property. Sci Rep.

[11] Wang L, Chen JL, Ge L, Zhu ZH, Rudolph V (2011). Halloysite nanotube supported Ru nanoparticles for ammonia catalytic decomposition to produce Co_x_-free hydrogen. Energy Fuel.

[12] Wang RJ, Jiang GH, Ding YW, Wang Y, Sun XK, Wang XH, Chen WX (2011). Photocatalytic activity of heterostructures based on TiO_2_ and halloysite nanotubes. ACS Appl Mater Interfaces.

[13] Liang J, Fan ZY, Chen S, Ding SJ, Yang G (2014). Hierarchical NiCo_2_O_4_ nanosheets@halloysite nanotubes with ultrahigh capacitance and long cycle stability as electrochemical pseudocapacitor material. Chem Mater.

[14] Zhang Y, Xie Y, Tang A, Zhou Y, Ouyang J, Yang H (2014). Precious-metal nanoparticles anchored onto functionalized halloysite nanotubes. Ind. Eng. Chem. Res..

[15] Kim M, Kim JK, Park JH (2015). Clay nanosheets in skeletons of controlled phase inversion separators for thermally stable Li-ion batteries. Adv Funct Mater.

[16] Cargnello M, Johnston-Peck AC, Diroll BT, Eric W, Datta B, Damodhar D, Doan-Nguyen VVT, Herzing AA, Kagan CR, Murray CB (2015). Substitutional doping in nanocrystal superlattices. Nature.

[17] Nogami M, Suzuki K (2002). Fast spectral hole burning in Sm^2+^-doped Al_2_O_3_-SiO_2_ glasses. Adv Mater.

[18] Jin J, Fu L, Ouyang J, Yang H (2013). 3D ordered macro-mesoporous indium doped Al_2_O_3_. Cryst Eng Comm.

[19] Yin H, Zhang CZ, Liu F, Hou YL (2014). Hybrid of iron nitride and nitrogen-doped graphene aerogel as synergistic catalyst for oxygen reduction reaction. Adv Funct Mater.

[20] Foresti E, Hochella MF, Kornishi H, Lesci IG, Madden AS, Roveri N, Xu HF (2005). Morphological and chemical/physical characterization of Fe-doped synthetic chrysotile nanotubes. Adv Funct Mater.

[21] Levard C, Masion A, Rose J, Doelsch E, Borschneck D, Olivi L, Chaurand P, Dominici C, Ziarelli F, Thill A, Maillet P, Bottero JY (2011). Synthesis of Ge-imogolite influence of the hydrolysis ratio on the structure of the nanotubes. Phys Chem Chem Phys.

[22] White RD, Bavykin DV, Walsh FC (2012). Spontaneous scrolling of kaolinite nanosheets into halloysite nanotubes in an aqueous suspension in the presence of GeO_2_. J Phys Chem C.

[23] Yue HR, Zhao YJ, Zhao S, Wang B, Ma XB, Gong JL (2013). A copper-phyllosilicate core-sheath nanoreactor for carbon-oxygen hydrogenolysis reactions. Nat Commun.

[24] Liang XL, Lin ZY, Yang YX, Xing ZW, Chen GR (2012). Luminescence properties of Tb-Eu Co-doped aluminosilicate and zinc silicate glasses. J Am Ceram Soc.

[25] Čapková P, Matějka V, Tokarský J, Peikertová P, Neuwirthová L, Kulhánková L, Beňo J, Stýskala V (2014). Electrically conductive aluminosilicate/graphene nanocomposite. J Eur Ceram Soc.

[26] Wang P, Shen BJ, Shen DD, Peng T, Gao JS (2007). Synthesis of ZSM-5 zeolite from expanded perlite/kaolin and its catalytic performance for FCC naphtha aromatization. Catal. Commun..

[27] Ariga K, Lvov YM, Kawakami K, Ji QM, Hill PJ (2011). Layer-by-layer welf-assembled shells for drug delivery. Adv Drug Deliv Rev.

[28] Lvov YM, Wang WC, Zhang LQ, Fakhrullin R (2016). Halloysite clay nanotubes for loading and sustained release of functional compounds. Adv Mater.

[29] Jeong G, Kim YU, Krachkovskiy SA, Lee CK (2010). A nanostructured SiAl_0.2_O anode material for lithium batteries. Chem Mater.

[30] Yang Y, Liang QQ, Li JH, Zhuang Y, He YH, Bai B, Wang X (2011). Ni_3_Si_2_O_5_(OH)_4_ multi-walled nanotubes with tunable magnetic properties and their application as anode materials for lithium batteries. Nano Res..

[31] Duarte HA, Lourenço MP, Heine T, Guimarães L (2012) Clay mineral nanotubes: stability, structure and properties. Stoichiometry and Materials Science - When numbers matter. InTech

[32] Margraf JT, Ruland A, Sgobba V, Guldi DM, Clark T (2013). Theoretical and experimental insights into the surface chemistry of semiconductor quantum dots. Langmuir.

[33] Joussein E, Petit S, Churchman J, Theng B, Righi D, Delvaux B (2005). Halloysite clay minerals-a review. Clay Miner.

[34] Choi J, Park H, Hoffmann MR (2010). Effects of single metal-ion doping on the visible-light photoreactivity of TiO_2_. J Phys Chem C.

[35] Zhu H, Du ML, Zou ML, Xu CS, Fu YQ (2012). Green synthesis of Au nanoparticles immobilized on halloysite nanotubes for surface-enhanced raman scattering substrates. Dalton Trans.

[36] Zhang Y, Fu L, Yang H (2012). Insights into the physicochemical aspects from natural halloysite to silica nanotubes. Colloids Surf. A.

[37] Jin J, Fu L, Ouyang J, Yang H (2015). Carbon hybridized halloysite nanotubes for high-performance hydrogen storage capacities. Sci Rep.

[38] Niu M, Yang H, Zhang X, Wang Y, Tang A (2016). Amine-impregnated mesoporous silica nanotube as an emerging nanocomposite for CO_2_ capture. ACS Appl Mater Interfaces.

[39] Shu Z, Zhang Y, Yang Q, Yang H (2017). Halloysite nanotubes supported Ag and ZnO nanoparticles with synergistically enhanced antibacterial activity. Nanoscale Res Lett.

[40] Jin J, Ouyang J, Yang H (2017). Pd nanoparticles and MOFs synergistically hybridized halloysite nanotubes for hydrogen storage. Nanoscale Res Lett.

[41] Vuong NM, Reynolds JL, Conte E, Lee Y (2015). H:ZnO nanorod-based photoanode sensitized by CdS and carbon quantum dots for photoelectrochemical water splitting. J Phys Chem C.

[42] Sun H, Zhao P, Zhang F, Liu Y, Hao J (2015). Ag_2_S/CdS/TiO_2_ nanotube array films with high photocurrent density by spotting sample method. Nanoscale Res Lett.

[43] Tian F, Hou D, Hu F, Xie K, Qiao X, Li D (2017). Pouous TiO_2_ nanofibers decorated CdS nanoparticles by SILAR method for enhanced visible-light-driven photocatalytic activity. Appl Surf Sci.

[44] Kim YK, Park H (2011). Light-harvesting multi-walled carbon nanotubes and CdS hybrids: application to photocatalytic hydrogen production from water. Energ Environ Sci.

[45] Jia L, Wang DH, Huang YX, Xu AW, Yu HQ (2011). Highly durable N-doped graphene/CdS nanocomposites with enhanced photocatalytic hydrogen evolution from water under visible light irradiation. J Phys Chem C.

