# Supplementary material for: Substitutional Doping for Aluminosilicate Mineral and Superior Water Splitting Performance
Source: Nanoscale Res Lett. 2017 Jul 14;12:456. doi: 10.1186/s11671-017-2192-8 (PMC5511126; doi:10.1186/s11671-017-2192-8)
Supplement: Additional file 1: — Supporting Information for Substitutional Doping for Aluminosilicate Mineral and Superior Water Splitting Performance. (DOCX 3550 kb) [file 11671_2017_2192_MOESM1_ESM.docx]

**Additional file 1**

**Substitutional Doping for Aluminosilicate Mineral and Superior Water Splitting Performance**

**By Yi Zhang ^†, *^,** **Liangjie Fu ^†^, Zhan Shu, Huaming Yang^*^, Aidong Tang,** **Tao Jiang**

Dr. Y. Zhang, Dr. L. Fu, Z. Shu, Prof. T. Jiang, Prof. H. Yang
Centre for Mineral Materials

School of Minerals Processing and Bioengineering

Central South University

Changsha 410083 (China)

* Email: yee_z10@csu.edu.cn , hmyang@csu.edu.cn

Dr. Y. Zhang, Dr. L. Fu, M. Long, Prof. H. Yang
Key Laboratory for Mineral Materials and Application of Hunan Province

Central South University

Changsha 410083 (China)

Prof. H. Yang

State Key Laboratory of Powder Metallurgy

Central South University

Changsha 410083 (China)

Dr. A. Tang
School of Chemistry and Chemical Engineering

Central South University

Changsha 410083 (China)

^†^ These authors contributed equally to this work.

**Table S1**  Lattice constants of HNTs and La-HNTs samples

| Samples |  | Lattice plane | | | | | | |
| --- | --- | --- | --- | --- | --- | --- | --- | --- |
|  |  | (001) | (100) | (002) | (110) | (003) | (210) | (300) |
| HNTs | 2Theta | 11.90 | 19.91 | 24.46 | 34.93 | 38.11 | 54.41 | 62.46 |
|  | d(Å) | 7.42 | 4.45 | 3.64 | 2.57 | 2.36 | 1.69 | 1.49 |
| La-HNTs | 2Theta | 11.79 | 19.86 | 24.37 | 34.78 | 37.88 | 54.50 | 62.36 |
|  | d(Å) | 7.50 | 4.46 | 3.65 | 2.58 | 2.37 | 1.68 | 1.49 |

**Table S2** Positions and assignments of the IR vibration bands for HNT and La-HNT samples

| Position (cm^-1^) | Assignments | Position (cm^-1^) | Assignments |
| --- | --- | --- | --- |
| 3700 | O-H stretching of inner-surface hydroxyl groups | 909 | O-H deformation vibration of inner Al-OH groups |
| 3623 | O-H stretching of inner hydroxyl groups | 553 | Deformation of Al-O-Si |
| 1641, 1825 | Deformation of adsorbed water. | 467 | Deformation of Si-O-Si |
| 1040 | Skeleton Si–O–Si stretching vibration |  |  |

**Table S3** Porous textures of HNTs and La-HNTs

| Samples | BET surface area (m^2^/g) | BJH Pore volume (cm^3^/g) | Average Pore Size (nm) |
| --- | --- | --- | --- |
| HNTs | 82.58 | 0.41 | 19.85 |
| La-HNTs | 59.22 | 0.37 | 25.23 |

**Table S4** Comparison of photocatalytic H_2_ evolution rates of CdS-based photocatalysts

| Type of material | Hydrogen Evolution (μmol·h^-1^·g^-1^) | Reference |
| --- | --- | --- |
| CdS | 115, 40 | [43,45] |
| CdS/TiO_2_ | 129 - 678 | [43] |
| CdS/CNTs | 60 - 370 | [44] |
| CdS/N-graphene | 10 - 210 | [45] |
| CdS/HNTs | 260 | Present work |
| CdS/La-HNTs | 475 | Present work |

CdS-based composite photocatalysts are synthesized by the similar method. Typically, supporters were firstly dissolved into Cd^2+^ solution, and then dissolved into S^2-^ solution. Sample content is 100 mg.

**Table S5** Atomic concentration of different elements for HNTs and La-HNTs samples

| Samples | Atomic concentration (%) | | | | Al/Si ratio |
| --- | --- | --- | --- | --- | --- |
|  | O 1s | Al 2p | Si 2p | La 3d |  |
| La-HNTs | 61.67 | 14.34 | 23.03 | 0.96 | 0.62 |
| HNTs | 66.29 | 15.73 | 17.97 | - | 0.88 |


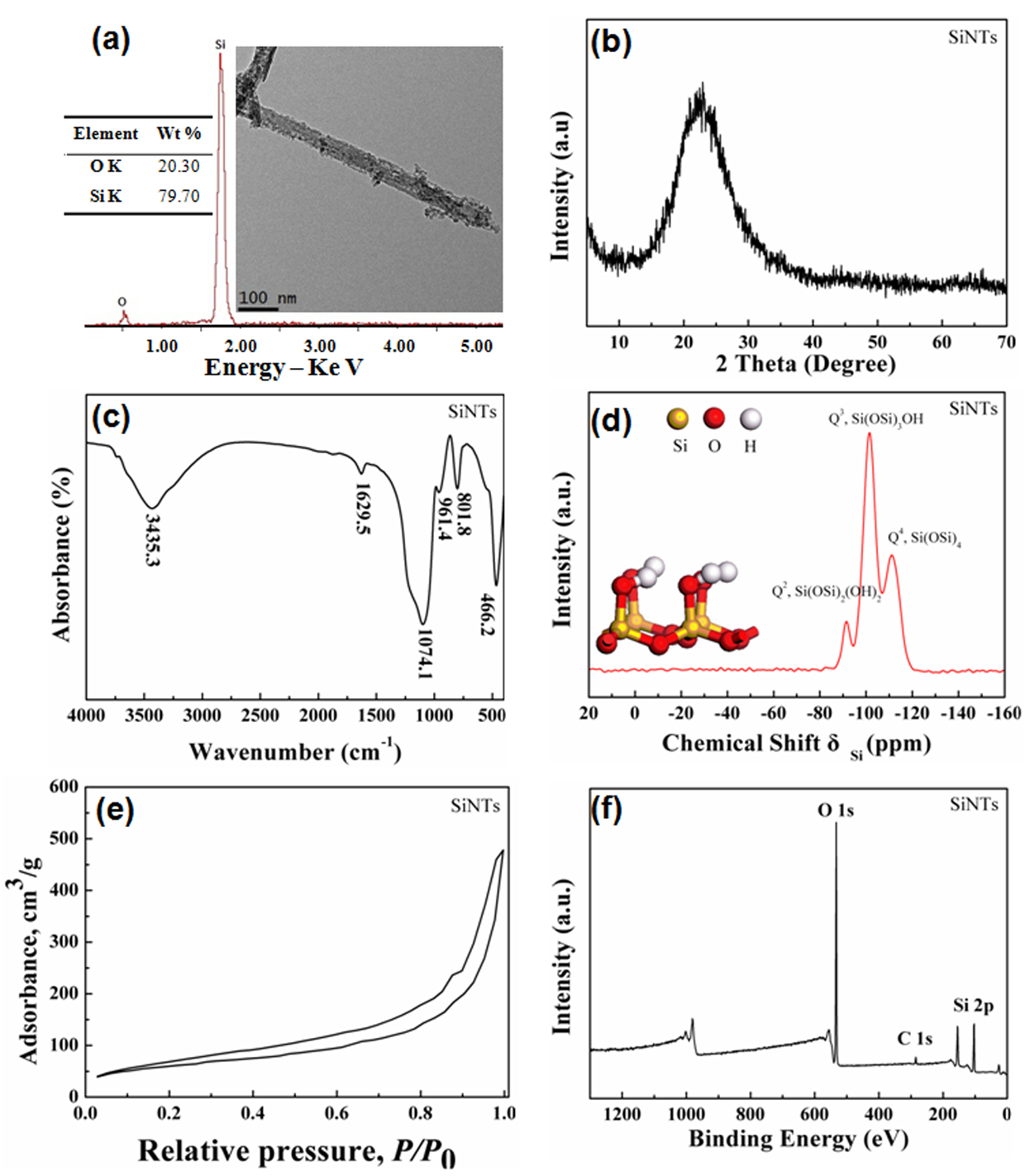


**Figure S1** (a) TEM image and corresponding EDS spectrum, (b) XRD patterns, (c) FTIR spectra, (d) Si NMR spectra, (e) BET isotherms and (f) XPS spectrum of acid treated HNT (SiNT).


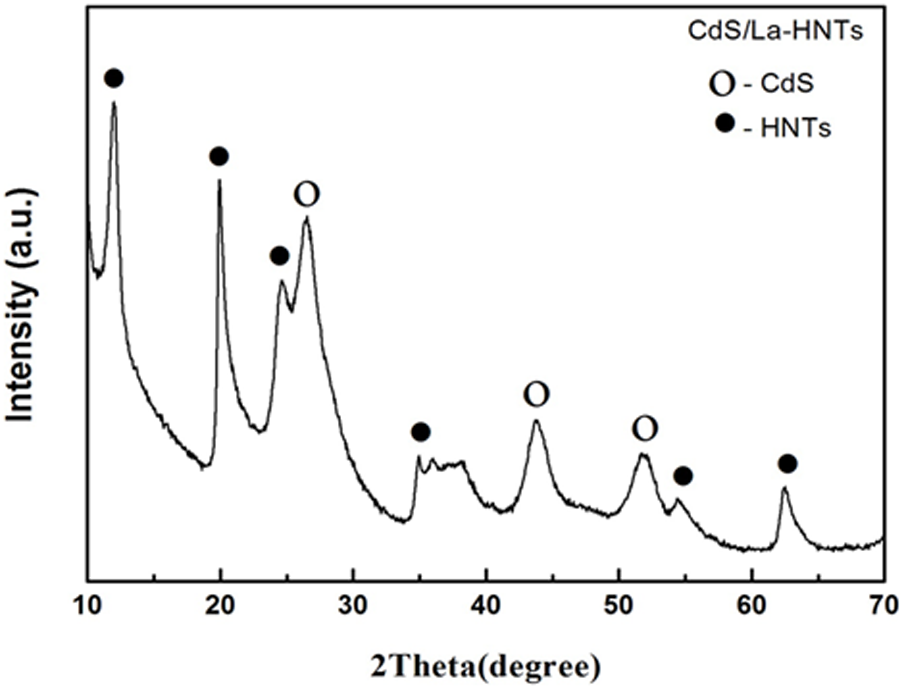


**Figure S2** XRD pattern of CdS/La-HNTs.


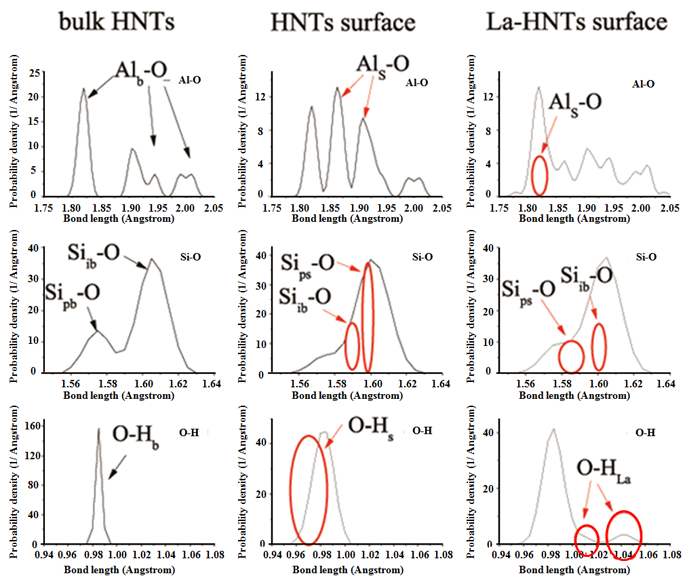


**Figure S3** The relaxed bond distributions for bulk HNTs, HNTs surface, and La-HNTs surface structures are given. (a) Al-O bond in HNTs bulk structure (Alb-O), and HNTs surface structure (Als-O). (b) Si-O bond perpendicular to the planar layer (Sipb-O or Sips-O) and in the planar layer (Siib-O or Siis-O) in HNTs bulk structure or in HNTs surface structure. (c) O-H bond in HNTs bulk structure (O-Hb), and HNTs surface structure (O-Hs). Surface OH bond with H heading direct to the OH of La(OH)_3_ (HLa).


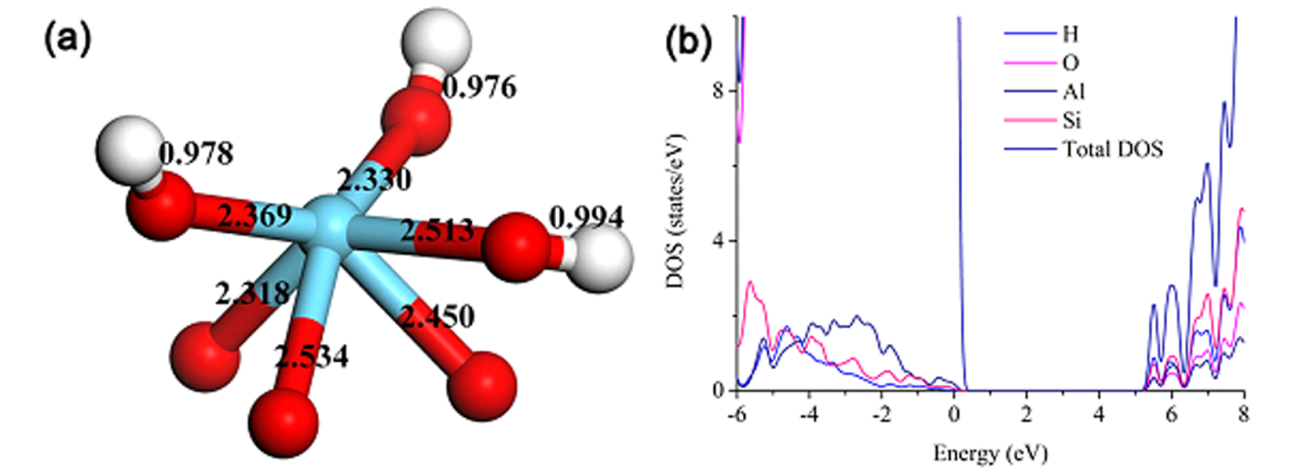


**Figure S4** (a) Calculated local structure around La-HNTs and (b) The PDOS result of HNTs surface.
